# Nanocontainer designed from an infectious hypodermal and hematopoietic necrosis virus (IHHNV) has excellent physical stability and ability to deliver shrimp tissues

**DOI:** 10.7717/peerj.6079

**Published:** 2018-12-18

**Authors:** Pauline Kiatmetha, Charoonroj Chotwiwatthanakun, Pitchanee Jariyapong, Wanida Santimanawong, Puey Ounjai, Wattana Weerachatyanukul

**Affiliations:** 1 Department of Anatomy, Faculty of Science, Mahidol University, Ratchathewi, Bangkok, Thailand; 2 Mahidol University, Nakhonsawan Campus, Mueng, Nakhonsawan, Thailand; 3 School of Medicine, Walailak University, Thasala District, Nakhonsrithammarat, Thailand; 4 Centex Shrimp, Faculty of Science, Mahidol University, Ratchathewi, Bangkok, Thailand; 5 Department of Biology, Faculty of Science, Mahidol University, Ratchathewi, Bangkok, Thailand

**Keywords:** IHHNV, Viral-like particles, VLP, Plasmid DNA, Encapsidation, Delivery system

## Abstract

**Background:**

A virus-like particle (VLP) is an excellent tool for a compound delivery system due to its simple composition, symmetrical structure and self-assembly. Its surface modification both chemically and genetically is established, leading to the target-specific delivery and improved encapsulation efficiency. However, its physical stabilities against many harsh conditions that guarantee long term storage and oral administration have been much less studied.

**Methods:**

IHHNV-VLPs were reconstructed from recombinant IHHNV capsid protein in *E. coli*. Their physical properties against three strong physical conditions including long term storage (0–30 days) in 4 °C, physical stabilities against broad ranged pH (4–9) and against three types of digestive enzymes were tested. Disassembly and reassembly of VLPs for encapsidating an enhanced green fluorescent protein tagged plasmid DNA (EGFP-VLPs) were controlled by the use of reducing agent (DTT) and calcium specific chelating agent (EGTA). Lastly, delivering ability of EGFP-VLPs was performed *in vivo* by intramuscular injection and traced the expression of GFP in the shrimp tissues 24 hr post-injection.

**Results:**

Upon its purification, IHHNV-VLPs were able to be kept at 4 °C up to 30 days with only slight degradation. They were very stable in basic condition (pH 8–9) and to a lesser extent in acidic condition (pH 4–6) while they could stand digestions of trypsin and chymotrypsin better than pepsin. As similar with many other non-enveloped viruses, the assembly of IHHNV-VLPs was dependent on both disulfide bridging and calcium ions which allowed us to control disassembly and reassembly of these VLPs to pack EGFP plasmid DNA. IHHNV-VLPs could deliver EGFP plasmids into shrimp muscles and gills as evident by RT-PCR and confocal microscopy demonstrating the expression of GFP in the targeted tissues.

**Discussion:**

There are extensive data in which capsid proteins of the non-enveloped viruses in the form of VLPs are constructed and used as nano-containers for therapeutic compound delivery. However, the bottleneck of its application as an excellent delivery container for oral administration would rely solely on physical stability and interacting ability of VLPs to the host cells. These properties are retained for IHHNV-VLPs reported herein. Thus, IHHNV-VLPs would stand as a good applicable nanocontainer to carry therapeutic agents towards the targeting tissues against ionic and digestive conditions via oral administration in aquaculture field.

## Introduction

Recently, many studies have focused on nano-containers designed from non-enveloped viral capsid proteins, so-called virus-like particles (VLPs). Specifically, their capability of encapsulating therapeutic cargoes, either protein-based or nucleotide-based, have rendered them potentially powerful tools for many advanced applications (Abbing et al., 2004; Douglas & Young, 1998; Varpness et al., 2005). Moreover, recent advances in genetic and chemical modifications of the capsid surfaces, both exteriorly and interiorly, have led to the target-specific delivery as well as an improved encapsulating efficiency in the more controllable fashion (Gillitzer et al., 2002; Kaltgrad et al., 2008). Owing to their structural simplicity, natural viral capsids are usually made up of one or a few capsid structural proteins which allow them to form nano-sized particles in a structurally symmetrical manner. In addition, viral capsids selectively interact with the host cell membrane and subsequently internalize into the cells, mostly via receptor-mediated endocytosis (Conner & Schmid, 2003; Pelkmans & Helenius, 2003). The reconstructed VLPs exhibit similar characteristics as those infectious virions, i.e., self-assembly to form nano-sized particles and cell receptor recognition (Ludwig & Wagner, 2007; Uchida et al., 2007). With such versatile properties, many attempts have directed towards manipulating VLPs to display foreign epitopes that serve as building blocks for carrying heterogeneous genetic materials to attack many diseases.

Extensive documents have been reported about reconstruction and applications of VLPs that are designed from both plant and animal non-enveloped icosahedral viruses (Martelli et al., 2011; Potera, 2012; Thiéry et al., 2006; Yusibov et al., 2002). However, applications of shrimp virus-derived VLPs to fight against many shrimp diseases are still limited (Jariyapong et al., 2015a), although there has been hard hits of shrimp aquaculture industries by several viral infections (Flegel, 2012). It is thus important that shrimp virus-derived VLPs with an inherent property to recognize shrimp tissues should be developed together with the RNAi technology for fighting against many shrimp viral infections. This is particular to many serious viral infections that greatly affect shrimp growth and mortality which cause a severe loss in shrimp farming, such as white spot syndrome virus (WSSV), infectious hypodermal and hematopoietic necrosis virus (IHHNV), yellow head virus (YHV) and many others (Lightner, 2011). An attempt to utilize RNAi machinery to halt shrimp viral infection in the aquaculture field has been launched (Feng et al., 2017; Saksmerprome et al., 2009). However, delivery of RNAi into shrimp tissues for viral treatment is still limited (Itsathitphaisarn et al., 2017). The advanced treatment through packaging of dsRNA into VLPs derived from *Macrobrachium rosenbergii* noda virus-like particle (MrNV-VLPs) has been in a laboratory trial and paved a way for shrimp viral infection halting (Jariyapong et al., 2015a; Jariyapong et al., 2015b). It should be noted that several excellent physical properties mentioned above also hold true for shrimp virus-derived VLPs. Additionally, the superior physical properties of shrimp viral VLPs are their stability against many harsh physical conditions which are reported for MrNV-VLPs (Jariyapong et al., 2014) and might also hold true in the case of other shrimp viruses. Therefore, we chose to further extrapolate the physical properties of recombinant IHHNV-VLPs and investigate their abilities to encapsidate and deliver a large-sized EGFP tagged-plasmid DNA *in situ* towards shrimp tissues.

## Materials and Methods

### Molecular cloning and purification of IHHNV capsid proteins

The IHHNV virus capsid gene was amplified from ORF3 of the IHHNV virus genome (with an estimated size of 990 bp). The specific primers for IHHNVcap (F: 5′-CATATGTGCG CCGATTCAACAAGAGCAA-3′ and R: 5′-CTCGAGTTAATGATGATGATGATGATGGTT AGTATGCATAACATAACATTTG-3′) were designed and used to amplify the gene using a gradient PCR method. The reaction conditions of PCR were one cycle at 94 °C for 5 min, followed by one set of 94 °C (30 s), 52 °C (1 min), and 72 °C (1 min) with a final extension step at 72 °C (10 min). The PCR product was ligated into pGEM-T Easy cloning vector (Promega, Madison, WI, USA), and transfected into *Escherichia coli* (*E. coli*). After screening, the positive clones were transfected into pET-17b expression vector (Novagen, Darmstadt, Germany) for protein expression in *E. coli* - Rosetta strain. The crude *E. coli* extracts were purified by a Ni-NTA affinity chromatography (Qaigen, Hilden, Germany), eluted by an imidazole-based elution buffer and dialyzed against Tris buffer saline (TBS; pH 7.8) as described earlier (Jariyapong et al., 2014). The purified proteins were stored at −80 °C with addition of protease inhibitor. These IHHNV-VLPs in TBS were used for all experiments below.

### Stability tests of the IHHNV capsid proteins

We tested the physical stability of IHHNV capsid proteins against three separated conditions including (1) long-term storage at 4 °C for 1–90 days; (2) a broad range of pH (4–9); and (3) digestion by gastrointestinal enzymes (trypsin, chymotrypsin and pepsin). Approximately 1 mg/ml of IHHNV-VLPs were re-suspended in TBS buffer (pH 7.8) and the suspension was allowed to stand in the refrigerator (4 °C) for the periods of 1 day, 30 days, and 90 days. Degradation of the purified protein band was monitored by SDS-PAGE and silver staining. For a broad pH range tests, the protein pellets after ultracentrifugation were re-suspended in either citrate buffer (pH 4.0 and adjusted to pH 5 and 6 by 1N NaOH) or in TBS (pH 7.8 and adjusted to the desired pH by 1N HCl or 1N NaOH). The VLP samples were maintained in the given pH at 4 °C for 24 hr and the reaction was stopped by adding a loading buffer into the samples and the protein profiles were resolved by SDS-PAGE as mentioned below.

For enzymatic digestion tests, IHHNV capsid proteins were divided into four groups as follows: (1) 1:50 (v/v) cocktail inhibitor (as negative control); 2 and (3) 30 mU trypsin and chymotrypsin in TBS, pH 7.8 (Sigma, St. Louis, MO, USA); and 4) 30 mU pepsin in citrate buffer, pH 4.0 (Sigma). The mixtures were allowed to stand for 1 h at room temperature and the enzymatic reaction was stopped by adding SDS-PAGE loading buffer into the mixtures. All testing samples were denatured at 95 °C and then resolved by 12.5% polyacrylamide gel electrophoresis (SDS-PAGE) followed by a silver staining.

### Ultra-structures of disassembled-reassembled IHHNV-VLPs and their encapsulation of EGFP tagged plasmid DNA

Disassembly and reassembly of VLPs was followed the protocol described previously by  Xing et al. (1999) with some modifications (Jariyapong et al., 2014). Briefly, the VLPs were incubated in a disassembling buffer (50 mM Tris-HCl, pH 7.5, containing 1 mM ethylene glycol tetraacetic acid (EGTA), 20 mM dithiothreitol (DTT), and 150 mM NaCl) for 1 hr at room temperature. To reassemble the VLPs, a reassembling buffer (containing 5 mM CaCl_2_ in TBS) was added at 10-time volume of the disassembling buffer, followed by incubation at room temperature for 1 hr. Both disassembled and reassembled IHHNV-VLPs were washed and collected using an ultracentrifugation (246,000× g, 4 °C, 2 h). The VLP pellets were then re-suspended in TBS and dropped onto a 200-mesh, carbon activated, formvar- coated grid (EMS, PA, USA) for 30 s. The excess suspension was blotted away by filter paper and the adhered VLPs were stained with 1% uranyl acetate for 30 s followed by visualization under a FEI Tecnai 20 transmission electron microscope operated at 80 kV.

In order to encapsidate a large sized Pie1-DNA vector tagged with an enhanced green fluorescent protein (EGFP), we followed DNA encapsidation protocol reported earlier for IHHNV-VLPs (Hou et al., 2009) with some modifications (Jariyapong et al., 2014). Approximately 10 µg of IHHNV-VLPs were disassembled as mentioned above and an equal amount of 3.1 kbp plasmid DNA vector was added into the suspension. They were allowed to stand for 1–2 h in the room temperature. Thereafter, a reassembling buffer was added at 10-time volume of VLP suspension, allowed to stand for 4–5 h, and ultracentrifuged to collect VLPs as mentioned earlier. The efficiency of plasmid DNA vector encapsidation was checked by 1% agarose gel electrophoresis followed by ethidium bromide staining.

### SDS-PAGE and silver staining

SDS-PAGE was performed according to Laemli’s method (1970). The following sets of proteins were resolved: (1) 10 µg of crude extracts from *E. coli* cells with or without (for cell incubation) protease inhibitor cocktail (1:50 (v/v)); (2) 400 ng of the purified IHHNV-VLPs; (3) IHHNV-VLPs storing at 4 °C for 1–90 days; (4) IHHNV-VLPs subjected to a broad range of pH (4–9) treatments; and (5) IHHNV-VLPs treated with 30 mU trypsin, chymotrypsin and pepsin. All proteins were heated for 5 min at 95 °C. The denatured proteins were resolved in a 12.5% SDS-PAGE under a reducing condition. The separated proteins were subjected to silver staining using a kit from Invitrogen (Thermo Fisher Scientific, Waltham, MA, USA).

### Administration and detection of IHHNV-VLPs in the shrimp tissues

Animal handling protocol was followed the guidelines of the Animal Care Committee, Mahidol University (reference no. MU-IACUC 2016/013). Approximately 10 µg in 100 µl of the IHHNV-VLPs encapsulating Pie1-EGFP plasmid DNA (Borirak et al., 2009) were injected intramuscularly into 3 gm Pacific white shrimp, *P.vanamei* (*n* = 15 shrimp/group). One-day post injection (p.i.), the administered shrimp were anesthetized on iced water until no movement was detected. Shrimp tissues including gill, muscle and hepatopancreas were collected and analyzed for the presence of EGFP-plasmid DNA in the shrimp tissues via RT-PCR as well as detected for the fluorescent signals under confocal microscopy. In an alternative experiment, the antibody against GFP was also used to verify the presence of GFP in the tissues of administered shrimp. For RT-PCR, pair of primers used to detect GFP gene were (F) 5′-CATGGTCCTGCTGGAGTTCGTG-3′ and(R) 5′-CGTCGCCGTCCAGCTCGACCAG-3′. The PCR amplification condition was followed the PCR steps mentioned above.

For confocal microscopy, the tissues of the VLP administered shrimp were fixed with 4% paraformaldehyde, immersed in 30% sucrose and solidified in a Sakura TissueTek medium (Alphen an den Rijn, Netherlands). After freeze-plunging in liquid nitrogen, the tissues were sectioned at 20 µm, blocked with 5% BSA, 0.1% Tween 20 in TBS and subsequently stained with 1:5,000 rabbit anti-GFP (GenScript, Piscataway, NJ, USA) in the blocking solution. The tissues were further exposed to 1:2,000 goat anti-rabbit IgG conjugated with Alexa593 (Molecular Probes, Eugene, OR, USA) and counterstained with 4′, 6-diamidino-2-phynylindole dihydrochloride (DAPI; DNA staining dye) and visualized with an Olympus FV10i confocal laser scanning microscope. Image acquisition was performed in a Kalman line-by-line scanning mode using laser diode (LD) lines (excitation wavelengths = 405, 478 nm and 635 nm, respectively). The emission filters used was the 5-nm band pass filters with wavelengths of 460 nm (blue fluorescence), 520 nm (green fluorescence) and 630 nm (red fluorescence).

Proteins from the tissues of the shrimp, both injected with EGFP-IHHNV-VLPs or unloaded IHHNV-VLPs, were extracted by a lysis buffer (1 mM EDTA, 1% Triton X-100, 0.1% SDS, 1 mM PMSF, 10 mM Tris-Cl, pH 8.0). Protein concentration was measured by a Bradford’s protein assay kit (Sigma, St. Louis, MO, USA) and subjected to SDS-PAGE as mentioned above. The resolved proteins were transferred to nitrocellulose membrane and subjected to anti-GFP probing in the same conditions described for confocal microscopy (except the secondary antibody that was replaced by goat anti-rabbit IgG conjugated with horse radish peroxidase. The enzymatic product was visualized by an enhanced chemiluminescence (ECL) kit and developed on a Hyperfilm (Amersham Pharmacia, Buckinghamshire, UK).

## Results

### Production of recombinant IHHNV capsid protein

**Figure 1:**
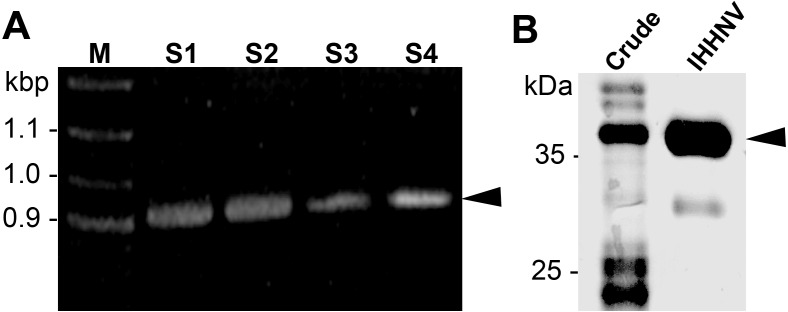
IHHNV insert and recombinant protein.

The IHHNV capsid protein was designed from ORF3 of IHHNV gene leading to a 990 bp construct with six histidine (His) flanking at the C-terminus (Fig. 1A). After inserted into the pET17b plasmid, we chose to express the recombinant proteins in *E. coli* (Rosetta) which has been shown to facilitate the expression of capsid proteins within 24 h yielding 2–3 mg/ml of the purified IHHNV capsid proteins. Through a single-step Nikel affinity chromatography, a highly purified (>95% purity) IHHNV capsid protein at the molecular mass of 37.5 kDa was obtained from the crudely prepared *E. coli* extracts (Fig. 1B). A minor band of 30 kDa represented a low level of degradation of purified VLP.

### Physical stability of IHHNV against harsh conditions

**Figure 2:**
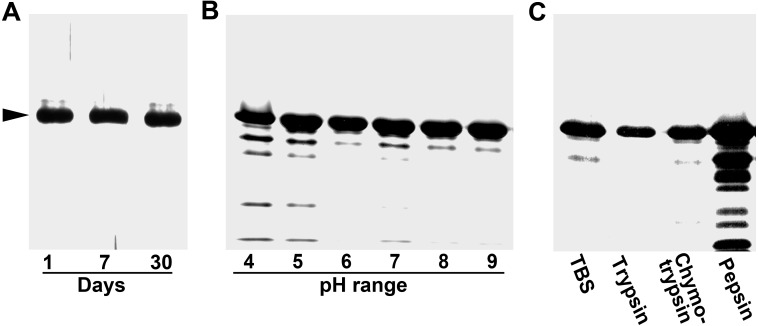
VLP stability test. Physical stability tests of IHHNV-VLPs against long term storage (A); acidic and basic conditions (B); and strong digestive conditions (C).

We further tested the physical properties of IHHNV-VLPs against three harsh conditions including long-term storage in a cold atmosphere, pH gradient, and strong hydrolytic milieu (mimicking gastrointestinal environment in digestive system). Generally, our results demonstrated that the readily formed IHHNV-VLPs were very stable upon their storage in TBS suspension at 4 °C. The 37.5 kDa band in SDS-PAGE appeared relatively constant among all samples studied (Fig. 2A, arrowhead), suggesting a high stability of IHHNV-VLPs when they were kept in the form of suspension.

For the physical stability against a broad range of pH, it was apparent that IHHNV-VLPs had a very strong protein tolerance against basic condition, pH 7 up to pH 9 (Fig. 2B), a much stronger basic condition than that of the milieu in small intestine. Correspondingly, VLP stability tests with the enzymes present in the intestinal digestive juice, trypsin and chymotrypsin, showed relatively small hydrolytic effect over the protein integrity of IHHNV-VLPs—less than 5% of the capsid proteins were degraded into the 30 kDa band for both enzymes (Fig. 2C). IHHNV-VLPs were also largely stable in a mild acidic condition (pH 6), where the 37.5 kDa capsid protein band intensity was rather constant. The VLPs became more susceptible to the strong acidic condition of pH 4–5 in the citrate buffer in which about 10–12% of proteins were degraded. The degradable protein bands of 30, 25, 15 and 10 kDa were clearly visible (Fig. 2B). The strongest VLP hydrolytic effect was observed with pepsin enzyme digestion (whose optimal function is at pH 4.0). In this case, more than 50% of the 37.5 kDa band was digestible into many smaller protein bands when pepsin was included into the VLP suspension (Fig. 2C). The structural stability of IHHNV-VLPs towards three different harsh conditions was summarized in Table 1.

**Table 1:** Stability of IHHNV-VLPs in the three different testing conditions.

**Percent stability**	**Storage at 4 °C**	**pH range**	**Enzyme tests**
≥97	1–7 days	6–9	Trypsin
≥95	30 days	5	Chymotrypsin
≥85	–	4	–
<80	–	–	Pepsin

**Figure 3:**
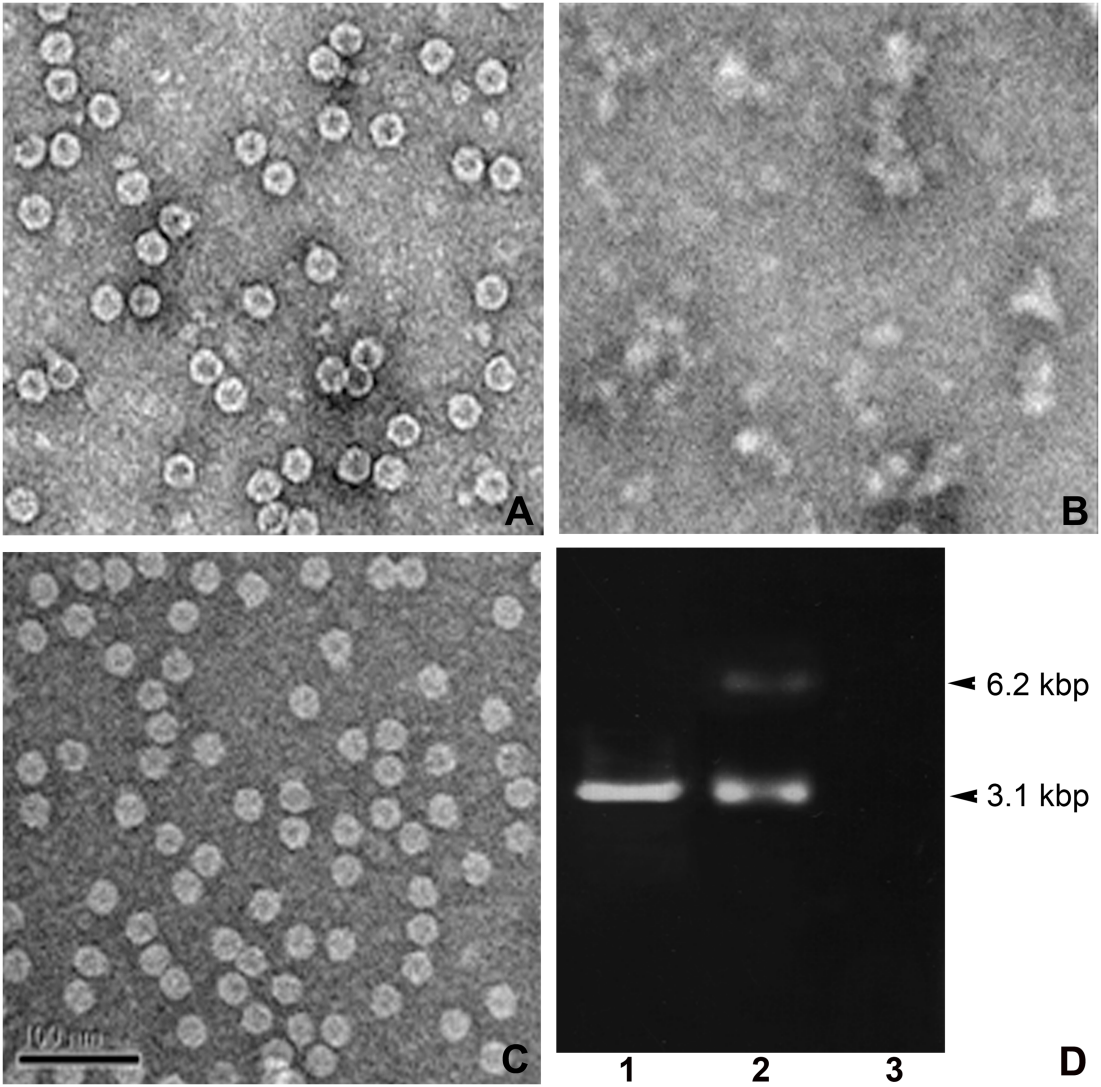
TEM structures of VLPs and encapsidation. Effect of combined calcium (EGTA) and reducing agent (DTT) in IHHNV-VLP disassembly/reassembly (A–C) and encapsidation of EGFP-plasmid DNA into IHHNV-VLP interior at 3.1 kbp (D).

### Control of disassembly and reassembly of IHHNV-VLPs

The other important physical property of VLP that renders it an applicable nano-container is the controllable VLP disassembly/reassembly. It has been reported that IHHNV-VLPs expressed *E. coli* BL21 (DE3) possess a property to encapsidate the host-cell derived genomic DNA and RNA (400–600 bp) into the VLP’s cavity (Hou et al., 2009). We further modified the test of this VLP’s property to encapsulate a plasmid DNA tagged with the traceable green fluorescent reporter which would allow us to follow VLP targeting in the shrimp tissues. In addition, as the exogenous calcium ions have been proven to be essential for shrimp virus assembly (Jariyapong, 2015), we thus included it into the encapsidation process to facilitate and control VLP disassembly/reassembly. Figure 3A demonstrated the mulberry-like structural feature with the diameter of about 27 nm of the untreated IHHNV-VLPs as reported earlier (Hou et al., 2009). However, the inner cavity of these bacterial derived VLPs showed an empty or very few electron dense materials, suggesting less genomic contaminant in the VLPs expressed in *E. coli* (Rosetta). In the presence of 10 mM EGTA (a calcium specific chelator) together with DTT, the VLPs became highly dispersed, disorganized and clumped in some areas—there was no mulberry-like structure remained in all EM visualizing fields that we scanned (Fig. 3B). With the addition of 5 mM CaCl_2_ into the suspension, the normal mulberry-like structure of VLP was recovered and the aggregated clumps became evenly distributed particles in the suspension (Fig. 3C). In addition, inclusion of 10 µg EGFP-plasmid DNA into the disassembled VLPs followed by their reassembly (with CaCl_2_) successfully encapsidated plasmid DNA into the VLP cavity which was revealed by the presence of 3.1 kbp band. The unloaded plasmid often showed a minor band of 6.2 kbp which assumed to be the dimerized complex of vector DNA (lane 2). It should be mentioned here that non-encapsidated VLPs showed no nucleotide band (lane 3), confirming a minimal nucleotide contamination in IHHNV-VLPs prepared from this *E. coli* strain as mentioned above. Our results thus implicate that inclusion of calcium ions into the suspension eases the control of VLP disassembly and reassembly processes which, in turns, facilitate the encapsidation of tagged plasmid DNA into the VLP interior.

### Targeting of IHHNV-VLPs in the shrimp tissues

**Figure 4:**
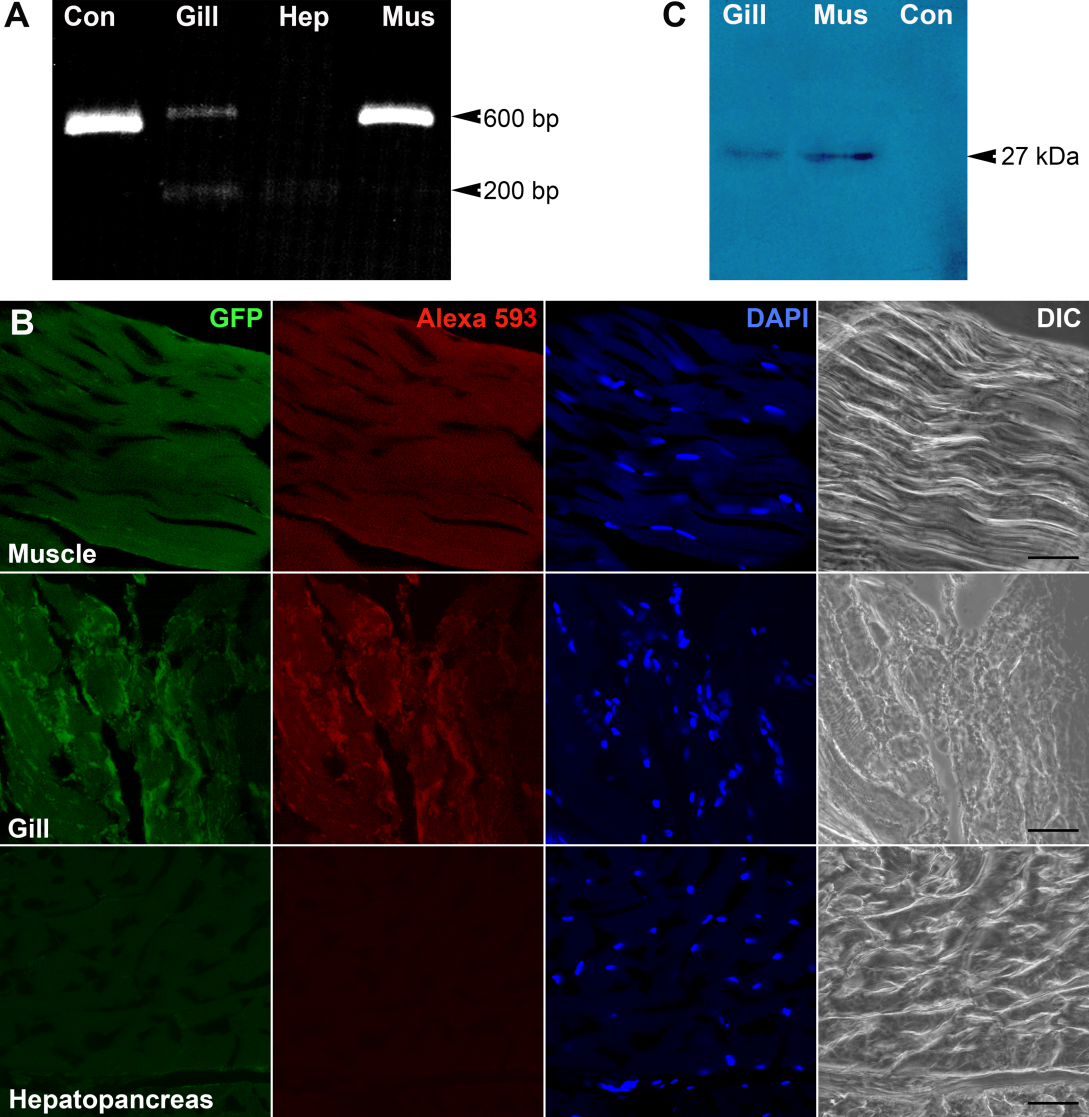
Tissue targetting of EGFP-VLPs. Detection of EGFP upon administration of IHHNV-VLPs encapsidating EGFP-plasmid DNA in the challenged shrimp tissues by RT-PCR (A), confocal microscopy (B) and Western blotting probed with anti-GFP (C).

We utilized IHHNV-VLPs encapsidating Pie1-EGFP as a testing delivery tool to investigate the potential targets of this virus in the Pacific white shrimp, *P. vannamei*. Upon 24-hr administration of EGFP-VLPs into the shrimp, we could detect a positive PCR product of GFP gene (600 bp) in the gill and muscular tissues of the injected shrimp (Fig. 4A, arrowhead). Hepatopancreas tissues did not show any positive PCR band at this 600 bp position suggesting that at least two tissues of *P. vanamei,* gill and muscles, that appeared to be IHHNV infection targets.

We further confirmed IHHNV infectivity in the target tissues by confocal microscopy (Fig. 4B). An intense green fluorescent signal of EGFP expression was clearly detected in the muscle fibers taken from thoracic segment (‘Muscle’ row). Likewise, gill tissues at lateral body wall also exhibited moderate to intense fluorescence in the radial fibers throughout the tissues studied (‘Gill’ row). Presence of GFP in the tissues was also verified by anti-GFP staining both by confocal microscopy revealing an intense red fluorescence (second column of each row) and Western blotting revealing a single immunostaining band at 27 kDa representing GFP protein (Fig. 4C). Hepatopancreas exposed to EGFP-IHHNV-VLPs (bottom row) or the thoracic muscles of shrimp exposed to non-encapsidated IHHNV-VLPs (control; see https://figshare.com/articles/Kiatmetha_Raw_Data_3_4/6809318) showed minimal green fluorescence in the tissues –only the blue fluorescence of DNA staining were detected (‘Hepatopancrease’ row).

## Discussion

It has been reported by Hou et al. (2009) that IHHNV capsid proteins are able to express in *E. coli* (BL21-DE3) at 16 °C for 48 hr which spontaneously form icosahedral VLPs. They have also observed that the expressed IHHNV-VLPs could encapsidate *E. coli* derived genomic DNA or some RNA materials (with the size of 400–600 bp) into VLP interior. In this study, we used *E. coli* (Rosetta) to express IHHNV capsid proteins to further extrapolate the VLP physical stability against many harsh conditions as well as to encapsidate the larger sized (3.1 kbp) plasmid DNA tagged with EGFP. Moreover, the ability of IHHNV-VLPs to deliver EGFP-plasmid *in situ* towards shrimp tissues is one of the highlight in our findings. We found that production of recombinant IHHNV-VLPs in *E. coli* (Rosetta*)* offered somewhat faster production rate (within 24 h in LB broth) and relatively higher protein yield in the same culture period which has been used before for MrNV capsid protein production (Jariyapong et al., 2014). In addition, much less nucleotide contaminating materials in the capsid cavity appeared to be another advantageous aspect of IHHNV-VLPs expressed in this *E. coli* system (Fig. 3). With this superior structural feature of VLPs, we are now conducting cryo-TEM approach to further analyze the atomic structures of specific capsid domains through the design of several recombinant IHHNV-VLP variants without any disturbing factors from nucleotide contaminations. Most interestingly, IHHNV-VLPs expressed in this *E. coli* also possessed the intactness of viral interaction machineries on their surface topography that are necessary for host cell receptor binding. This claim was experimentally evident by the binding and internalization of IHHNV-VLPs into the host cells’ cytoplasm in both gill and muscular tissues of the IHHNV-VLPs administrating shrimp (Fig. 4).

The other excellent physical property that directly determines whether the VLPs can be applied through an oral administrative route is its ability to endure many harsh conditions within the alimentary tract environment. It was evident herein that IHHNV-VLPs were rather stable in the basic environment, similar to that of the luminal compartment of the small intestine. Even the VLPs were less stable in the acidic environment (comparable to the gastric environment); however, an appreciable amount of capsid proteins (about 95%) still remained after encountering the VLPs to the moderate and strong acidic conditions (pH 4–6). In addition, the VLPs were rather stable against trypsin and chymotrypsin which are two major constitutive digestive enzymes in the alimentary tract although it is less stable against pepsin. More interestingly, the stability of IHHNV-VLPs against long-term storage or having a slow spontaneous degradation over monthly storage would offer them an outstanding nano-container that is highly practical for field trials (Hooker, Kovacs & Francis, 2004). IHHNV-VLPs thus stand for a high opportunity towards a good industrialized model where the VLPs could be kept as a suspension in the refrigerator and is ready-mixed with the food pellets for oral administration or submersion treatment of larvae on-site in several aquaculture farm areas.

Binding that leads to internalization of the virions during their infection of many non-enveloped shrimp viruses is dependent on the specific protruding domain that is usually localized on the 5-fold axis of the capsid surface where the DE loops from the five subunits meet (Kaufmann et al., 2010; Somrit et al., 2016). It has also been reported that viral surface conformation may be altered and becomes architectural difference between the native viruses (filled with genome interiorly) and the empty recombinant VLPs. This information hold true in many cases of either alpha- or beta-nodaviruses including MrNV and grouper nervous necrosis virus (GNNV) (Zhou et al., 2016) where the capside organizations are changed from *T* = 1 (in the empty VLPs) to be *T* = 3 (in the native virions). This surface topological difference between intact virions and VLPs casts a doubt whether the configuration of protruding domain that is responsible for viral binding to the host receptor should be affected to some certain extent. One known structural-base mechanism that is associated with the alteration of capsid subunit organization is the interaction between nucleotide sequences with the nucleic acid binding domain (Lata et al., 2000) that is present universally in either terminus of capsid proteins depending on nodavirus species. This nucleotide-guide capsid structural conformation is also known to be related with capsid maturation process during viral assembly (Schneemann, 2006; Tu et al., 2015) which is without a doubt essential for viral binding/internalization during their infection. Despite this structural dissimilarity of the capsid surface, our results clearly revealed that binding leading to internalization of IHHNV-VLPs was still spare –an intense fluorescent signal of EGFP expression could be observed in both muscular and gill tissues upon IHHNV-VLP administration (Fig. 4). The explanation of this phenomenon could be due to the well-known adaptability of viruses to interact with the host cells, so called “highjacking” ability of the viruses through many alternative routes of entry on the host cell surfaces. These include ligand–receptor mediated endocytosis, non-specific macro-pinocytosis or even non-identified endocytotic or phagocytotic pathways (Brandenburg & Zhuang, 2007). Regardless of the mechanism how IHHNV-VLPs enter shrimp tissues, our results indicating the excellent physical stability of VLPs against many strong ionic and digestive conditions as well as their calcium-based encapsidation would open up the practicality of utilizing this nanocontainer in the form of suspension for oral administration in aquaculture field trails.

## Conclusion

We have reported here the excellent physical properties of recombinant IHHNV-VLPs beyond other known non-enveloped viruses derived VLPs. They could endure many strong ionic and digestive conditions that pave a way to their application as an oral administration-based nano-container. Their encapsidation of plasmid DNA and presumably other therapeutic materials could be controlled through the use of DTT + EGTA to regulate VLP disassembly and exogenous CaCl_2_ to regulate VLP reassembly. Finally, IHHNV-VLPs retained the virion surface inherent property of interacting with the shrimp tissues *in vivo*. Together, we believe that IHHNV-VLPs should now be another step closer to the field application as a ready-mix suspension for oral administration or submersion trials to fight against many viral infections.

## Additional Information and Declarations

### Data Availability

The following information was supplied regarding data availability: Weerachatyanukul, Wattana (2018): Kiatmetha_Raw Data. figshare. Fileset. https://doi.org/10.6084/m9.figshare.6809270.v1. Weerachatyanukul, Wattana (2018): Kiatmetha_Raw Data2. figshare. Figure. https://doi.org/10.6084/m9.figshare.6809315.v1. Weerachatyanukul, Wattana (2018): Kiatmetha_Raw Data 3&4. figshare. Fileset. https://doi.org/10.6084/m9.figshare.6809318.v2.
